# Case Report: Embedded broken needle in a preterm infant following neonatal blood sampling

**DOI:** 10.3389/fped.2026.1896280

**Published:** 2026-07-22

**Authors:** Syed Hussein Fathi Syed Mahmud Shahab, Zhun Shen Tan, Mohd Kamarul Safie, Shareena Ishak, Marjmin Osman, Azrina Syarizad Kuthubul Zaman

**Affiliations:** 1 Faculty of Medicine, Universiti Kebangsaan Malaysia, Kuala Lumpur, Malaysia; 2 Paediatric Surgery Unit, Department of Surgery, Faculty of Medicine, Universiti Kebangsaan Malaysia, Kuala Lumpur, Malaysia; 3 Department of Pediatrics, Faculty of Medicine, Universiti Kebangsaan Malaysia, Kuala Lumpur, Malaysia

**Keywords:** broken needle, case report, foreign body, iatrogenic, neonatal blood sampling, neonate, preterm infant, surgical removal

## Abstract

A “broken needle” is an improvised technique for neonatal blood sampling used to facilitate an already technical procedure. This involves removing the plastic hub from a standard hypodermic needle to enable direct capillary blood collection, therefore carrying significant safety risks if the needle is mishandled or improperly disposed of. We describe a 10-week-old preterm infant who was recently discharged from a long stay in the neonatal intensive care unit (NICU), referred to the paediatric surgical unit with a swollen, erythematous swelling over the left flank. On examination, a pointed foreign body was palpated, prompting a plain abdominal radiograph to be performed. This revealed a long, thin, needle-like object in the left flank. Urgent computed tomography (CT) confirmed a pin-like foreign body abutting the splenorenal fossa, with reactive inflammatory changes but without definite organ penetration or significant injury. The patient underwent abdominal wall exploration and surgical removal of a 3 cm needle with an uncomplicated postoperative course and full recovery. This case underscores an underrecognized but serious complication of this blood-sampling technique: an embedded needle with organ-adjacent foreign-body implantation requiring surgical retrieval. Clinicians should therefore adhere strictly to standard blood-taking protocols, use approved neonatal blood-sampling devices where available, and ensure proper staff training and safe disposal practices. Institutions with NICUs should also prioritize the availability of appropriate neonatal sampling equipment to prevent similar preventable incidents and safeguard patient safety.

## Introduction

Preterm infants require frequent blood sampling due to unstable clinical conditions and complications, leading to significant iatrogenic blood loss and increased transfusion needs (1). Given the challenges of performing repeated phlebotomy in these infants, some clinicians use a broken needle to facilitate blood collection. This technique involves removing the plastic hub from a standard hypodermic needle to enable direct blood flow into the collecting tube via capillary action. Modifying hypodermic needles for neonatal blood sampling is common in certain settings, including Brazilian neonatal units that have documented the use of the broken needle technique for peripheral venous puncture in preterm infants (2). These workarounds mean that medical devices are not always used as intended by manufacturers and may carry potential risks to the infant when mishandled or improperly discarded (3).

Neonatal blood sampling, especially in the neonatal intensive care unit (NICU), is increasingly recognized as a source of iatrogenic blood loss, with recent studies emphasizing the need to minimize unnecessary phlebotomy and optimize sampling protocols to reduce morbidity in preterm infants (1, 2). Against this backdrop, the use of unregulated improvised devices such as “broken needles” represents a hazardous deviation from standard practice. We report a case of a retained broken needle in a preterm infant following improper disposal during a blood sampling procedure, which ultimately required surgical removal. This case highlights a rare but serious and underrecognized complication of non-standard neonatal blood sampling techniques which underscores the need for strict adherence to established protocols and the use of approved devices to ensure patient safety.

## Case presentation

A 10-week-old female infant of Malay ethnicity, born at 28 weeks and 1 day gestation with a birth weight of 1.01 kg, was referred to the paediatric surgical unit for evaluation of a tender, erythematous swelling over the left flank. The infant was recently discharged from the NICU after a prolonged stay due to extreme prematurity at birth complicated by respiratory and metabolic derangements associated with prematurity.

**Figure 1:**
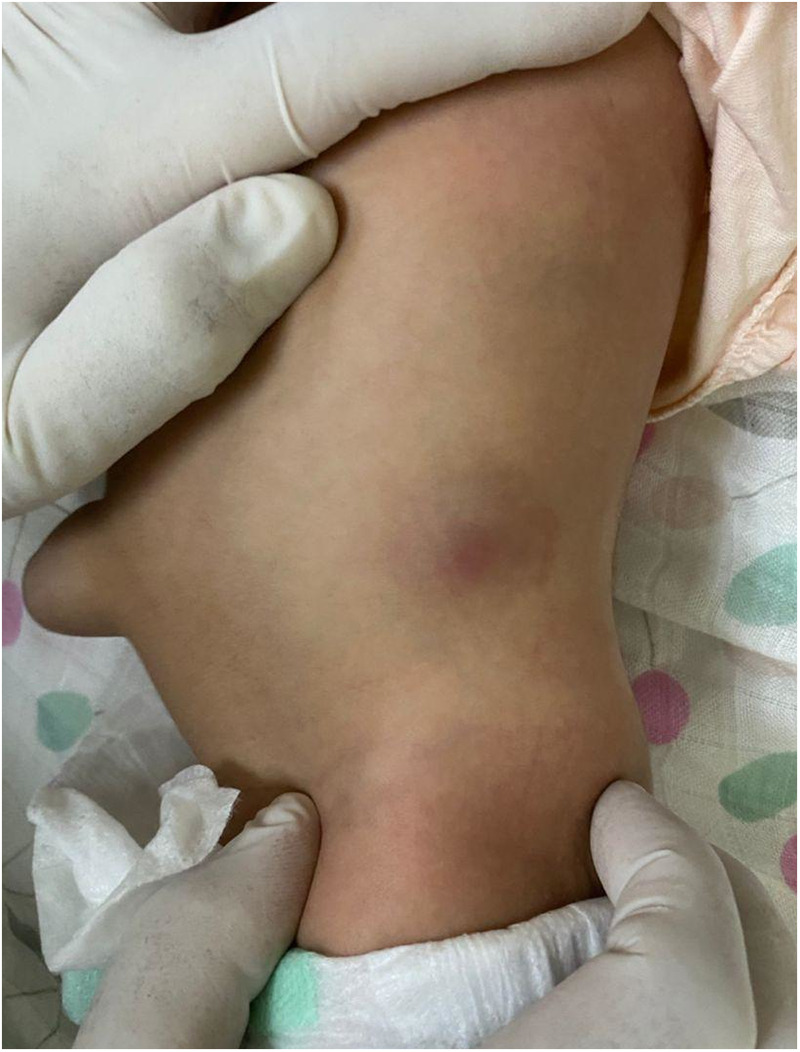
Appearance of the patient's left flank on initial assessment, demonstrating a localised soft-tissue swelling of approximately 2–3 cm in diameter with overlying erythema.

3 days after discharge, the infant presented with a painful, erythematous swelling over the left flank, accompanied by irritability. There were no systemic signs of sepsis, and the parents denied recent trauma or injection over the left flank. On examination, the infant was alert, afebrile, and haemodynamically stable. Examination of the left flank revealed a localised soft-tissue swelling approximately 2–3 cm in diameter with overlying erythema and a pointed, linear foreign body palpable beneath the skin (Figure 1). The overlying skin was intact without ulceration or sinus tract, and there was no surrounding crepitus or fluctuance. The rest of the physical examination was unremarkable, with no rashes, petechiae, respiratory distress, or abdominal signs beyond the left-flank lesion.

**Figure 2:**
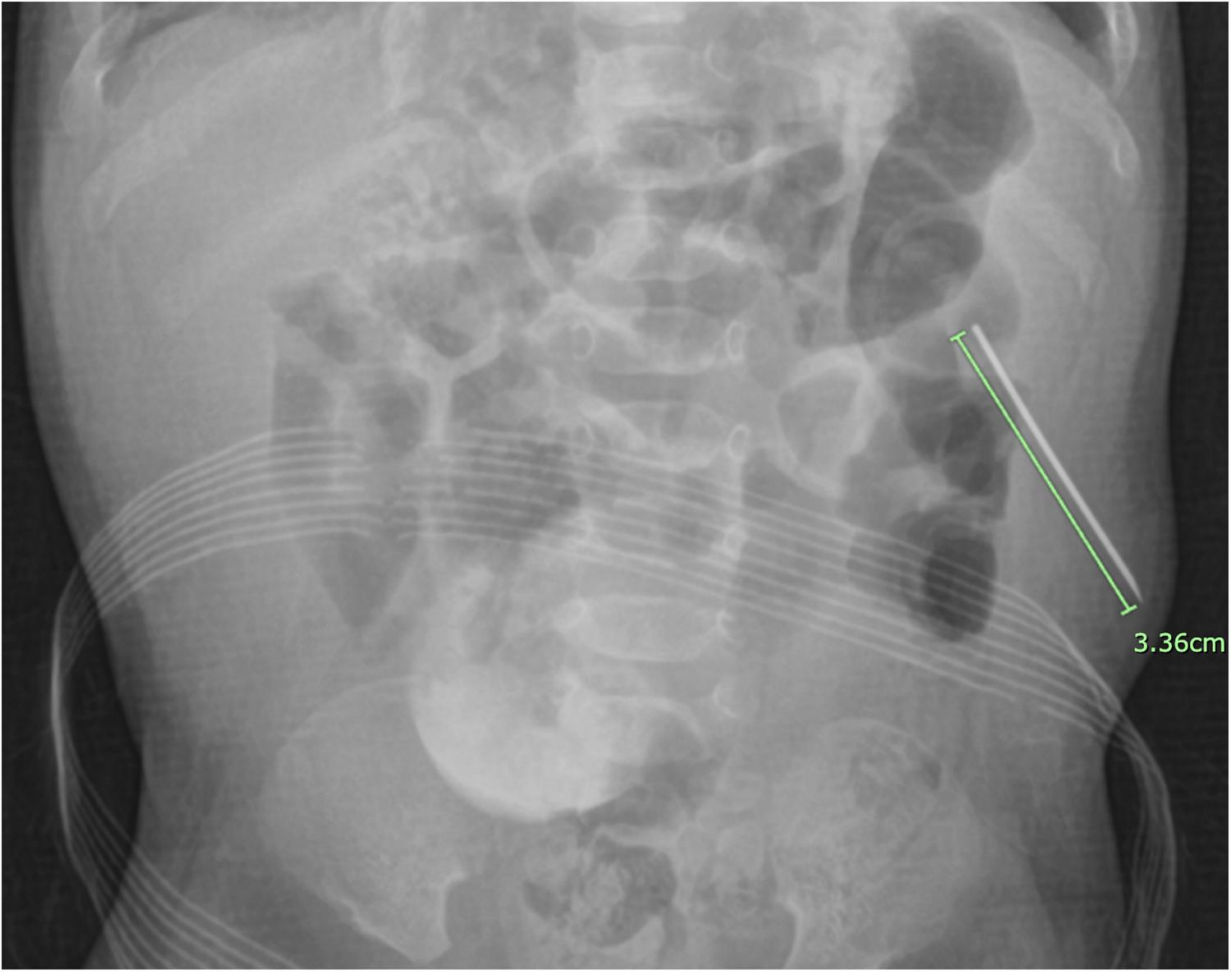
Plain abdominal radiograph demonstrating a thin, elongated needle-like foreign body oriented obliquely in the left flank, measuring 3.36 cm in length (green line).

**Figure 3:**
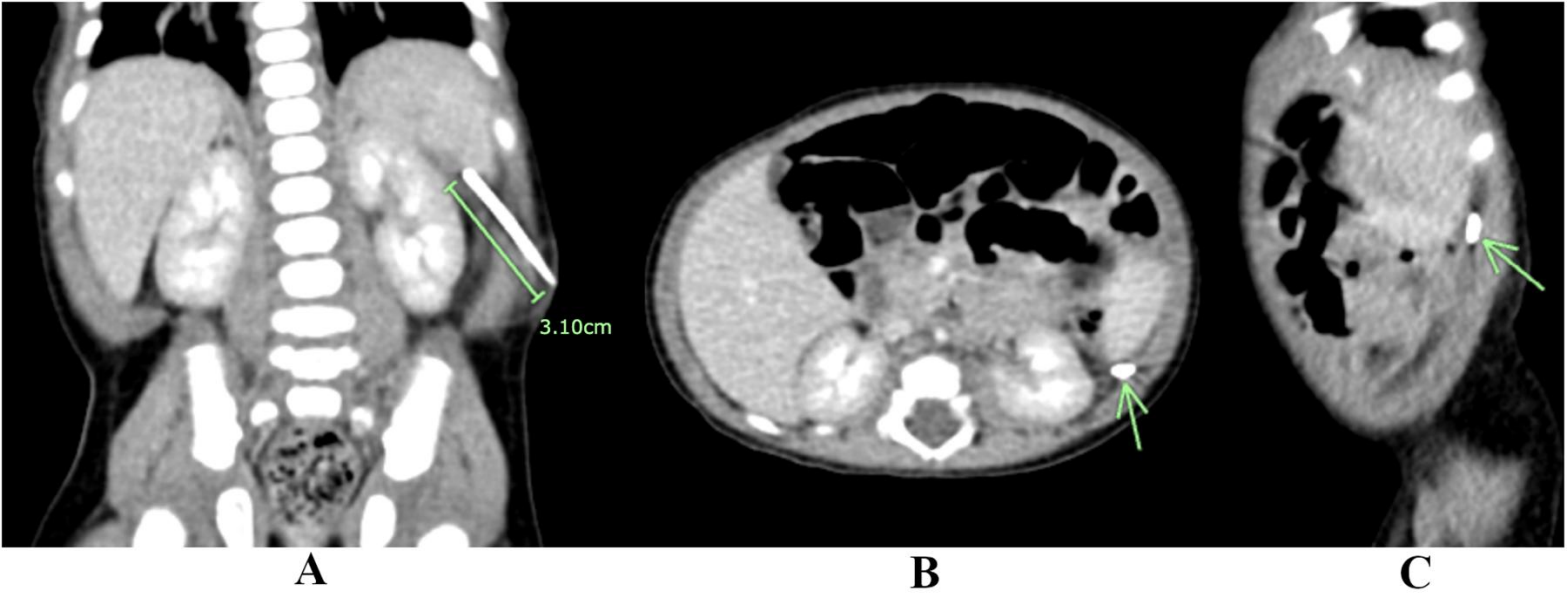
Computed tomography of the abdomen and pelvis. **(A)** Coronal view confirming a pin-like foreign body at the left flank measuring 3.1 cm (green line), with its blunt end extending to the splenorenal fossa. **(B)** Axial view demonstrating the blunt end of the foreign body (green arrow) in close proximity to the spleen, with associated reactive inflammatory changes in the subcutaneous tissues and minimal perisplenic fluid. **(C)** Sagittal view revealing the foreign body in cross-sectional profile (green arrow), confirming a lateral-to-medial trajectory of penetration through the left flank soft tissues.

Plain abdominal radiography revealed a thin, elongated needle-like foreign body oriented obliquely in the left flank, measuring 3.36 cm on formal radiographic measurement (Figure 2). An urgent contrast-enhanced computed tomography (CT) scan of the abdomen and pelvis was performed for further characterisation. Coronal view confirmed a pin-like foreign body at the left flank measuring 3.1 cm, with its blunt end extending to the splenorenal fossa (Figure 3A). Axial view demonstrated the blunt end of the foreign body in close proximity to the spleen (Figure 3B). Sagittal view revealed the foreign body in cross-sectional profile rather than in longitudinal axis (Figure 3C), confirming a lateral-to-medial trajectory of penetration through the left flank soft tissues. The CT images showed associated reactive inflammatory changes in the subcutaneous tissues and minimal perisplenic fluid; there was no evidence of internal organ injury, wall thickening, or rim-enhancing collection. Laboratory investigations, however, demonstrated no systemic evidence of infection or inflammation, with a white cell count of 7.8 × 10⁹/L, haemoglobin 11.1 g/dL, haematocrit 34.8%, and platelet count 290 × 10⁹/L, alongside a C-reactive protein of 0.45 mg/dL and procalcitonin of 0.14 ng/mL, both within normal limits. On this basis, the findings were interpreted as a localised embedded foreign-body reaction in close proximity to the spleen, likely iatrogenic in origin, without evidence of systemic sepsis or significant organ involvement.

**Figure 4:**
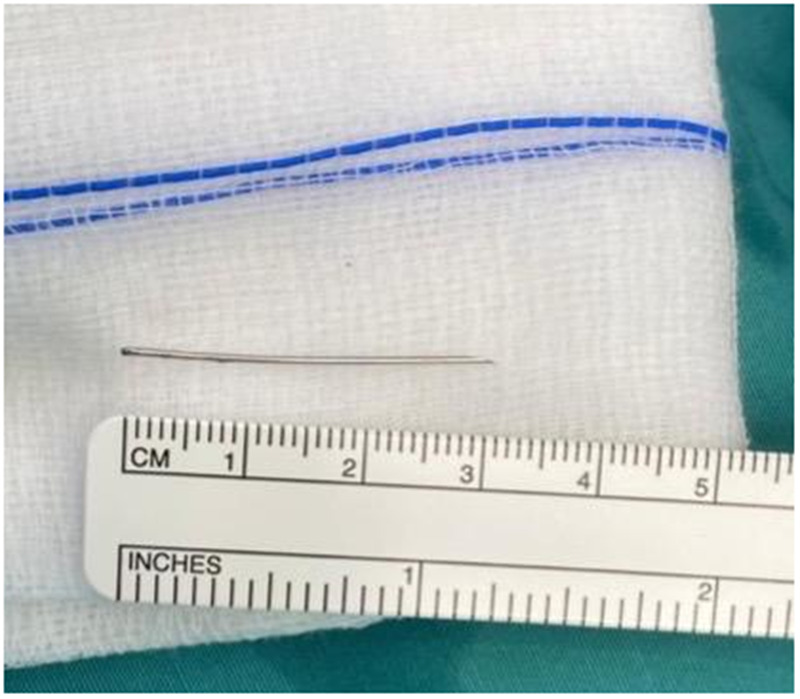
The retrieved broken needle measuring 3 cm in length, consistent with the foreign body identified on imaging.

The infant underwent skin and abdominal wall debridement with removal of the foreign body at the left lumbar region. Intraoperatively, a small incision was made overlying the skin where the pointed object was palpable. Minimal turbid inflammatory fluid was noted upon incision of the overlying skin and subcutaneous tissue following which a 3 cm needle-like foreign body with a bevelled tip (“broken needle”) was retrieved intact. (Figure 4). The surrounding muscle and fascia appeared macroscopically healthy without obvious fat necrosis or significant purulent infiltration. The wound was irrigated copiously and closed in layers. She tolerated the procedure well and was discharged with complete resolution of local swelling with no short-term complications. Follow-up at the neonatal clinic 6 months later revealed a well healed small 0.5 cm wound.

## Discussion

This case presents an embedded broken needle–related foreign-body abscess in a preterm neonate, with the needle located in the left flank abutting the splenorenal fossa but without visceral penetration. The device was almost certainly iatrogenic, resulting from a modified hypodermic needle used during neonatal blood sampling, in which the plastic hub was removed to allow direct capillary collection. This “broken needle” technique has been described as an improvised workaround to facilitate venepuncture in preterm infants, perceived by clinicians as less aggressive and associated with shorter collection times (2, 4). However, improvised needle modifications create a thin, unguided, hub-less needle that is easily lost or embedded, particularly in small neonates.

In neonates and infants, the relatively soft and pliable subcutaneous tissue may facilitate migration of sharp foreign bodies along tissue planes, and case reports of retained needles in children and infants show that such objects can come into close proximity with vital organs without immediate perforation or overt clinical signs (5). The reactive inflammatory changes and minimal perisplenic fluid seen on CT are consistent with a localized foreign-body–related abscess rather than acute organ injury, yet they underscore the potential for chronic inflammation, secondary infection, and late complications if the object were left *in situ*. General reports on retained needles support the principle of early recognition, prompt imaging-guided localization, and removal (surgical or interventional) to prevent long-term complications, even when visceral breach is not clearly evident (3).

In the broader literature, neonatal blood sampling is increasingly recognized as a major source of iatrogenic harm, not only through blood-volume loss but also via device-related complications and procedure-related stress (1, 6, 7). Repeated phlebotomy in extreme preterm infants can account for a substantial proportion of their total blood volume over the first weeks of life, exacerbating anaemia of prematurity and transfusion needs (1, 6–8). Amid these concerns, recent guidelines and safety alerts emphasize the use of appropriate neonatal-specific sampling devices, minimization of unnecessary blood draws, and strict sharps-handling and disposal protocols, explicitly warning against improvised techniques such as hub-breaking or broken needle practices (2, 9, 10). The present case adds a vivid, clinically instructive example to this literature: the embedded needle arose from exactly the kind of *ad hoc* improvisation that formal protocols seek to prevent, and its removal via surgical debridement highlights the cost of such practices in fragile neonates (2, 5, 11).

## Limitations

This single-case report has inherent limitations in generalizability and causality. It describes an isolated event from 1 institution and cannot quantify the incidence or risk magnitude of “broken-needle” practices in neonatal units more broadly. Detailed information about the exact circumstances of the needle's origin, who performed the blood sampling, how disposal protocols were followed, and whether device modification occurred intentionally or inadvertently is lacking due to retrospective reliance on caregiver report and chart review, which introduces potential recall and reporting bias. Imaging and intraoperative findings excluded visceral perforation at the time of surgery but cannot determine the precise timing or migration pathway of the foreign body, nor the long-term sequelae beyond the 6-month follow-up reported here. Finally, because institutional practices, device availability, and staff training vary widely, the applicability of recommended system-level interventions should be interpreted cautiously and ideally evaluated in larger observational studies or audits.

## Conclusion

The primary take home lessons of this case report are clear and urgent. The use of an improvised broken needle technique in neonatal blood sampling carries serious and avoidable risks, including embedded foreign bodies that can migrate along anatomical tissue planes and about vital organs. This event occurred in the context of repeated phlebotomy in a preterm infant, where the pursuit of successful sampling must never eclipse rigorous sharps safety. Clinicians should adhere strictly to standard blood-taking protocols, use approved neonatal sampling devices wherever possible, and enforce immediate, point-of-care disposal of all needles. Institutions caring for neonates, especially NICUs, must recognize that the embedding of a broken needle in a preterm infant is not a minor technical mishap but a serious iatrogenic complication that can be prevented through system-wide policies, staff training, and a culture of safety that prioritizes evidence-based practice over improvisation.

## Data Availability

The original contributions presented in the study are included in the article/Supplementary Material, further inquiries can be directed to the corresponding authors.
